# Tracheostomy as a Management Option After Listing for Pediatric Cardiac Transplantation

**DOI:** 10.1111/petr.70029

**Published:** 2025-01-21

**Authors:** Elisabeth Day, Deborah Cross, David Crossland, Jason Powell, Emma Simpson

**Affiliations:** ^1^ Paediatric Intensive Care Freeman Hospital Newcastle upon Tyne UK; ^2^ Department of Paediatric Cardiology and Cardiothoracic Surgery Freeman Hospital Newcastle upon Tyne UK; ^3^ Paediatric Ear, Nose and Throat Great North Children's Hospital Newcastle upon Tyne UK; ^4^ Translational and Clinical Research Institute, Faculty of Medical Sciences Newcastle University Newcastle upon Tyne UK

**Keywords:** pediatric cardiac transplantation, survival, tracheostomy

## Abstract

**Background:**

Children with end‐stage heart failure listed for cardiac transplantation may require mechanical ventilation and/or circulatory support whilst awaiting transplantation. A subgroup of these patients is unable to wean off mechanical ventilator support and undergo tracheostomy to enhance quality of life and allow de‐escalation of intensive care. There is limited evidence of the use of tracheostomy associated with pediatric cardiac transplantation. We describe outcomes to better inform future management of these patients.

**Methods:**

A single‐centre, retrospective study was performed, assessing all pediatric patients (< 18 years) listed for cardiac transplant from 2006 to 2017. We assessed background demographics and outcomes, including cardiac diagnosis, complications, insertion of ventricular assist device and survival. We identified patients who underwent tracheostomy after listing and compared this group with non‐tracheostomised patients.

**Results:**

Two hundred and eleven patients were listed for cardiac transplant, of whom 44 (21%) underwent tracheostomy after listing. The main indication for tracheostomy was failure to wean from mechanical ventilation (36%). Complications after tracheostomy included localized infection, granuloma, obstruction and hemorrhage, but were generally minor. Median time for tracheostomy decannulation was 75 days. When comparing tracheostomy versus non‐tracheostomy patients, there were no significant differences in age, weight or time to transplant. Survival was comparable between the non‐tracheostomy and tracheostomy groups at 1‐year, 97% versus 94% respectively.

**Conclusion:**

There is no evidence from our study that patients awaiting cardiac transplant who undergo tracheostomy have significant complications or reduced survival. Tracheostomy is usually a short‐term measure and should be considered in the management of children receiving prolonged ventilation around the time of cardiac transplantation.

## Graphical abstract

Tracheostomy after listing for pediatric cardiac transplantation is a safe adjunct to assist progress. We show minimal side effects and no difference in survival between those with tracheostomy and those without over the full follow‐up period.

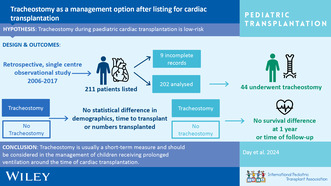

**Abbreviations**
ECMO: extracorporeal membrane oxygenation
IQR: interquartile range
LV: left ventricular
PIC: pediatric intensive care
VAD: ventricular assist device

## 1 Introduction

Children listed for cardiac transplantation are an increasingly complex group, often with significant comorbidities, who may require mechanical ventilation and/or circulatory support whilst awaiting or after transplantation. This can be for multiple reasons such as lung congestion, respiratory compromise and neurological deficit [1, 2, 3, 4]. Positive pressure ventilation in heart failure provides support by reducing left ventricular (LV) afterload and improving LV function as well as reducing work of breathing and therefore oxygen consumption [5]. However, prolonged mechanical ventilation also carries significant risk of morbidity including infection and long‐term airway complications, such as subglottic stenosis or vocal cord palsy [2, 6, 7]. Patients often require significant levels of sedation for safety which can lead to generalized muscle weakness as well as restricting their development over what can be a protracted period waiting for transplantation [2, 4].

Tracheostomy provides the opportunity to wake and mobilize these patients, improving their stability, sedation requirement and overall wellbeing [8]. It may also facilitate earlier discharge from intensive care. Some children on tracheostomy ventilation may be stable enough to be discharged from hospital and home milrinone is also an option. Children on a Berlin Heart ventricular assist device (VAD) tend to stay in hospital due to care needs and lack of approval for outpatient use. The use of tracheostomy is not without risk of morbidity and mortality [9, 10, 11], however there is increasing evidence in the general pediatric population of its effectiveness in patients requiring prolonged mechanical ventilation; reducing sedation requirements and increasing comfort [6, 9]. Previous studies have suggested that tracheostomy in pediatric patients after cardiac surgery may be associated with a higher risk of mortality than the general pediatric population [12, 13]. However, this is likely related to their underlying diagnoses, comorbidities and complex surgery, as opposed to the tracheostomy itself [2]. Morbidity associated with tracheostomy complications in this group has been shown to be low, encouraging increasing uptake in these patients where appropriate [2].

There are very few studies evaluating outcomes in patients undergoing tracheostomy around the time of cardiac transplantation [2, 4] and no UK studies of this group for 20 years. Our study is a single‐centre, retrospective analysis looking at the characteristics of patients requiring tracheostomy after listing for transplantation and comparing them with patients without tracheostomy to better understand this complex patient group and inform future management decisions.

## 2 Patients and Methods

### 2.1 Data Source and Definitions

Our hospital cardiac transplantation listing database was searched for all patients listed for cardiac transplantation. Data for the period of 1 January 2006 until 31 December 2017 were identified. Hospital electronic records were then used to identify patients who underwent tracheostomy and facilitate further analysis of all patients listed. A separate pediatric intensive care (PIC) database was searched for information regarding mechanical circulatory support using a VAD.

### 2.2 Study Population

All children under 18 years of age at time of listing for cardiac transplantation by the Pediatric Cardiology Department, Freeman Hospital, Newcastle upon Tyne, UK were eligible for inclusion.

### 2.3 Study Variables

Patient data were analyzed from cardiac transplant listing database for age, sex, weight at listing, time to transplantation and survival. Electronic patient records and scanned contemporaneous notes, including observation charts, were analyzed for patients who had undergone tracheostomy and then for variables including indication for tracheostomy, cardiac diagnosis, airway abnormalities, timing of tracheostomy, tracheostomy complications and time to decannulation. Cardiac diagnoses were categorized into four groups: acyanotic congenital cardiac lesion, cyanotic congenital cardiac lesion, single ventricle circulation and cardiomyopathy/myocarditis.

### 2.4 Study Endpoints/Outcomes

The primary outcome measure was survival after cardiac transplantation. Survival was measured at 30 days post‐listing, 1‐year post‐listing and at time of outcome follow‐up for the study on the 1st November 2024. After transplantation, survival was measured at PIC discharge, 30 days and 1‐year post‐transplant as well as at outcome follow‐up on the 1st November 2024. Secondary outcomes were duration of tracheostomy (time from tracheostomy insertion to decannulation) and early tracheostomy complications, taken at time of full cohort follow‐up on 31st March 2019, as well as comparison of survival and patient characteristics between these patients and those who did not require tracheostomy.

### 2.5 Statistical Analysis

Continuous variables such as age and weight are reported using median and interquartile range (IQR). Non‐continuous variables are reported using absolute number and percentage. Variables were analyzed using *T*‐test for continuous variables. Chi‐squared test, or Fisher's Exact test where group numbers were small, was used to compare non‐continuous variables with *p*‐value of < 0.05 considered statistically significant. Statistical analysis was performed using IBM SPSS 20 for Mac and Graphpad Prism 10.

Survival was analyzed using GraphPad Prism to produce Kaplan Meier charts and perform log rank test. When it was not known whether a patient was still surviving at the time of follow up, these patients were excluded from this analysis. A log rank test was used to compare survival between the two groups.

### 2.6 Ethical Approval

Approval for this study was received from Newcastle upon Tyne Hospitals NHS Foundation Trust Research and Development team. The need for ethical approval was waived.

## 3 Results

### 3.1 Overall

Our database search yielded 211 patients who were listed for cardiac transplantation between January 2006 and December 2017. Nine patients had incomplete records therefore could not be included in further analysis. Our cohort of 202 patients included 44 patients (22%) who underwent tracheostomy after listing, whilst 158 (78%) did not. Demographic information about the two groups is given in Table 1. Patients in both groups had broad IQR for weight and age at listing with a similar split between sex. Median weight was lower in the tracheostomy group (9.2 kg vs. 13.0 kg) whilst patients in this group were slightly younger at time of listing (28.4 months vs. 36.3 months). Eleven patients had genetic syndromes, these included Barth, Noonan's, Alstrom, Williams, 22q deletions and Senger's syndrome; of these patients, four underwent tracheostomy and seven did not.

**TABLE 1:** Demographics of tracheostomy and non‐tracheostomy patients.

	No tracheostomy (*n* = 158)				Tracheostomy (*n* = 44)				Significance (*p*)	
	*n*	(%)	Median	[IQR]	*n*	(%)	Median	[IQR]	*p*	
Male	84	(53)			19	(43)			0.241	
Weight (kg)			13.0	[7.6–26.2]			9.2	[6.4–20.3]	0.128	
Age at listing (months)			36.3	[8.5–104.6]			28.4	[9.0–138.0]	0.609	
Cardiac diagnosis									^a^	^b^
Acyanotic congenital cardiac lesion	12	(7)			5	(11)			0.538	0.038
Cyanotic congenital cardiac lesion	6	(4)			5	(11)			0.064	
Single ventricle circulation	39	(25)			16	(37)			0.130	
Cardiomyopathy/myocarditis	101	(64)			18	(41)			0.009	
Mechanical cardiac support										
ECMO	56	(35)			20	(45)			0.225	
VAD	78	(49)			24	(55)			0.543	
Any	99	(63)			32	(72)			0.216	

*Note:* This table describes demographics for tracheostomy patients and those who did not require tracheostomy. These demographics include sex, weight and age as well as cardiac diagnosis. The number of patients requiring ECMO or VAD support is also detailed for each subgroup as well as any mechanical cardiac support (note some patients received both ECMO and VAD support). *P* values are listed for significance of each comparison. There is a significantly lower chance of patients with cardiomyopathy/myocarditis requiring tracheostomy with *p* = 0.009 although numbers are small. Categorical variables are described as *n* (%). Non‐normally distributed continuous variables are described with median and interquartile range.

Abbreviations: ECMO, extra corporeal mechanical oxygenation; IQR, inter quartile range; *n*: number of patients; VAD, ventricular assist device.

^a^ Comparison of frequency of individual diagnosis performed using Fisher's Exact test due to small numbers in groups.

^b^ Comparison of frequency of all diagnoses performed using Chi Square.

Cardiac diagnoses are categorized for patients at the time of listing as well as the need for mechanical support over the study period (Table 1). Number of patients in each diagnostic category was small; however we note that a higher percentage of patients with single ventricle lesions made up the tracheostomy group (37% vs. 25%) and patients with cardiomyopathy/myocarditis were significantly less likely to require a tracheostomy (*p* < 0.01). Approximately half of tracheostomy patients (24/44; 55%) underwent both tracheostomy and VAD and 72% (*n* = 32) required either ECMO or VAD. Amongst non‐tracheostomy patients, 78 (49%) required VAD with 99 (63%) requiring some form of mechanical cardiac support. Tracheostomy did not reduce the likelihood of needing VAD pre‐transplant.

Patients were listed for transplantation for a median of 52 days (IQR 14–129) in the non‐tracheostomy group versus 47 days (IQR 15–75.5) in the tracheostomy group, see Table 2. There was no statistical difference between numbers transplanted, died on waiting list or delisted.

**TABLE 2:** Transplant Status.

	No tracheostomy (*n* = 158)		Tracheostomy (*n* = 44)		Significance (*p*)	
	*n*	(%)	*n*	(%)		
Status at follow up					^a^	^b^
Transplanted	126	(80)	33	(75)	0.5338	0.703
Delisted	12	(8)	5	(11)	0.5378	
Died awaiting transplant	20	(12)	6	(14)	0.8401	

	Median	[IQR]	Median	[IQR]	
Length of listing (days)	52	[14–129]	47	[15–75.5]	0.251

*Note:* This table details comparison between patients with and without tracheostomy and whether they underwent transplant, were delisted or died whilst still awaiting transplant at the time of follow‐up (1st November 2024). *P* values show there is no significant difference in status between patients with and without tracheostomy. Length of listing is given in days with no significant difference in median length of listing between those with and without tracheostomy although a broader IQR for those without tracheostomy. Categorical variables are described as *n* (%). Non‐normally distributed continuous variables are described with median and interquartile range.

Abbreviations: IQR, inter quartile range; *n*, number of patients.

^a^ Comparison of frequency of each status performed using Fisher's exact test due to small numbers in groups.

^b^ Comparison of frequency of all statuses performed using chi Square.

### 3.2 Tracheostomy Patients

**FIGURE 1:**
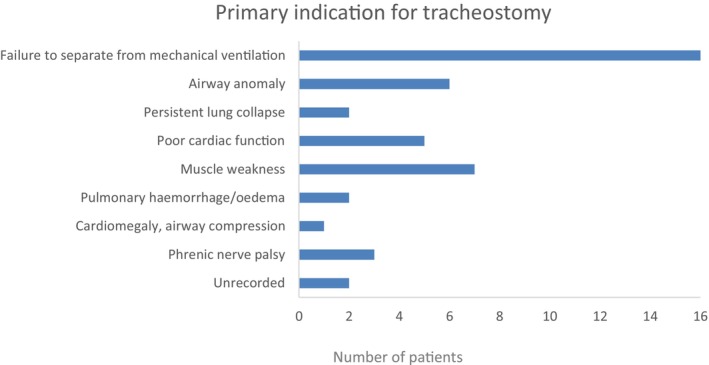
Indication for tracheostomy. Bar chart describing primary indication for tracheostomy as given in patient notes. Most commonly the indication for tracheostomy was described as failure to wean from ventilation or failed extubation. This highlights the complexity of these patients and the multi‐factorial nature of their failure to separate from mechanical ventilation.

Most tracheostomy patients underwent this procedure during the same hospital admission as their cardiac transplant with three identified as having tracheostomy pre‐listing. Indication for tracheostomy (Figure 1) was generally poorly defined. In 16 patients (36%), the only indication given was failure to extubate/wean from ventilation. Ten of the patients with “failure to extubate/wean from ventilation” as their only indication were noted to have some form of airway anomaly on bronchoscopy and three had phrenic nerve palsy. More specific indications were given for some patients including seven (16%) due to muscle weakness and six (14%) due to airway anomaly. The remainder included poor cardiac function, large heart (resulting in lung compression) and phrenic nerve palsy. Of those patients with specific indications identified (excluding “airway anomaly”), bronchoscopy revealed some form of airway anomaly in 11/19 patients. There were four patients where more than one reason for tracheostomy was given: one patient with muscle weakness and resultant lung collapse; one patient with muscle weakness and phrenic nerve palsy; one patient with large heart causing lung compression and phrenic nerve palsy; one patient with phrenic nerve palsy and recurrent pneumothoraces.

Early complications after insertion of tracheostomy were minor and infrequent with 27 (61%) patients having no reported issues. This is comparable with other literature [8, 14]. Complications included infection in nine patients, granuloma formation in five patients, minor bleeding in two patients and transient obstruction in two patients. Three patients had two separate complications with either granuloma and infection or granuloma and bleeding.

**FIGURE 2:**
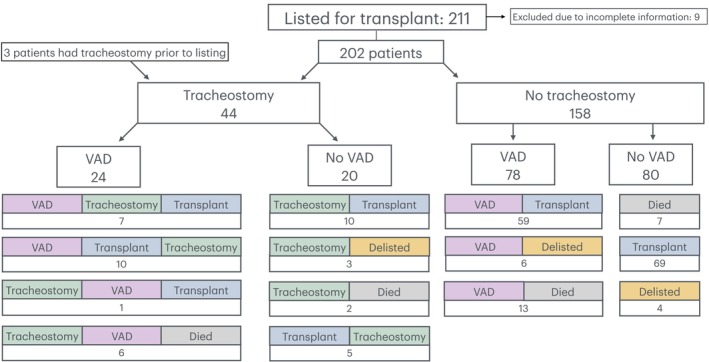
Chronology transplantation/tracheostomy/VAD. Figure showing the chronology of patient journey after listing for cardiac transplantation. This illustrates the division between tracheostomy and no tracheostomy and then subcategorises each of these groups according to whether they had Ventricular Assist Device (VAD) support. It also has colored boxes to represent the chronology of support/treatment given to each further subgroup of patients. These should be read from left to right in order of occurrence, for example 7 patients had VAD support followed by tracheostomy insertion and then transplantation. This is a complex figure with multiple subdivisions, emphasizing the complex journey these patients undergo.

The chronology of tracheostomy/VAD/transplantation was variable (Figure 2). Whilst the majority of patients (24, 55%) underwent tracheostomy before transplantation, 15 patients (34%) required tracheostomy after transplantation.

Patients were followed up until 31st March 2019 where tracheostomy was inserted. The median follow‐up was 2535 days (IQR 1144–3172 days, minimum 44 days, maximum 6683 days). Note, this excludes those who had tracheostomy prior to listing. Thirty two patients (73%) had their tracheostomy decannulated during the follow up period. Of those not decannulated, 9/12 died before this could be attempted (3 died prior to transplant, 3 died after transplant, 3 delisted and died), 2 patients continued with tracheostomy and 1 patient moved to another hospital therefore decannulation information was not available. Median time to decannulation was 75 days (IQR 38–453) and depended upon indication. The shortest period of tracheostomy was only 5 days in a patient with failure to wean from ventilation approximately 2 weeks after cardiac transplantation. The longest time to successful decannulation was 2331 days in a patient with single ventricle circulation who required a tracheostomy due to severe distal tracheomalacia and left main bronchomalacia.

### 3.3 Airway Anomalies

The majority of patients (139, 88%) who did not require tracheostomy did not have an airway examination. Of those who did, airway anomalies were documented in 16 patients, with no abnormality found in a further three patients who underwent bronchoscopy. Approximately two‐thirds of patients who required tracheostomy had an airway anomaly documented prior to tracheostomy (*n* = 28, 64%). Abnormalities found included upper airway obstruction, bronchomalacia and tracheomalacia with multi‐level abnormalities seen in five patients (11%). No abnormality was seen in 30% (*n* = 13) of these patients and three further patients had no recorded bronchoscopy.

### 3.4 Outcome

Patients were followed‐up for survival until 1st November 2024. This provides a median survival follow‐up period of 4310 days (IQR 1814–5194 days) with minimum follow‐up period 12 days and maximum 6745 days.

Tables 3 and 4 detail survival outcomes for both tracheostomy and no tracheostomy groups. One hundred and fifty‐nine patients underwent transplantation with the remaining patients not receiving transplant either due to clinical improvement, no organ availability or mortality. Details of survival outcomes for individual subgroups and statistical comparison is provided in Tables S1 and S2. Survival to 30 days post‐listing was 96%, survival to 1‐year post‐listing 96% and survival at time of follow‐up 75%. Comparing survival between tracheostomy versus non‐tracheostomy patients (regardless of whether they were ultimately transplanted), outcomes were similar and not statistically significant (Table 3). Finally, when comparing the cohort who did undergo transplantation with or without tracheostomy (Table 4), outcomes were also similar with survival to 30 days and 1‐year post‐transplant of 94% and 97% respectively then 73% and 76% respectively at time of final follow‐up.

**TABLE 3:** Survival—tracheostomy and no tracheostomy after listing.

	*n*	Survived to 30 days post listing		Survived to 1 year post listing		Surviving at follow‐up	
		*n*	(%)	*n*	(%)	*n*	(%)
Tracheostomy	44	41	(93)	33	(75)	26	(59)
No tracheostomy	158	143	(91)	125	(79)	105	(66)

Significance	*p*	0.5868		0.537		0.3514	
Relative risk (95% confidence intervals)		0.7 (0.2–2.4)		1.2 (0.7–2.2)		1.2 (0.8–1.9)	

All patients	202	184	(91)	158	(78)	131	(64)

*Note:* This table details survival for all patients after listing for cardiac transplantation as well as subdividing into survival for those with and without tracheostomy. It details survival at 30 days post‐listing, 1‐year post‐listing and survival at time of follow‐up (on 1st November 2024). It shows *p* values and 95% confidence intervals for each time period, comparing the two subgroups with no statistical difference seen between them.

Abbreviation: *n*, number of patients.

**TABLE 4:** Survival after cardiac transplantation.

	*n*	Survived to PIC discharge	Survived to 30 days post transplant	Survived to 1 year post transplant	Surviving at follow‐up
		*n* (%)	*n* (%)	*n* (%)	*n* (%)
Tracheostomy	33	31 (94)	31 (94)	31 (94)	24 (73)
No tracheostomy	126	123 (98)	122 (97)	122 (97)	96 (76)

Significance	*p*	0.2828	0.4434	0.4434	0.6769
Relative risk (95% confidence intervals)		2.6 (0.4538–14.9653)	1.9 (0.4–10.0)	1.9 (0.4–10.0)	1.1 (0.6–2.2)

All patients	159	154 (97)	153 (96)	153 (96)	120 (75)

*Note:* This table details survival for all patients who underwent cardiac transplantation as well as subdividing into those with and without tracheostomy. It details survival to PIC discharge, 30 days post‐listing, 1‐year post‐listing and survival at time of follow‐up (on 1st November 2024). It shows *p* values and 95% confidence intervals for each time period, comparing the two subgroups with no statistical difference seen between them.

Abbreviations: *n*, number of patients; PIC, pediatric intensive care.

## 4 Discussion

### 4.1 Overall

This study provides a detailed observational look at pediatric patients requiring tracheostomy after listing for cardiac transplantation. To our knowledge, this is the first UK study to look in detail at the use of tracheostomy around the time of pediatric cardiac transplantation and only the second international study [4] of this patient group.

### 4.2 Outcome

**FIGURE 3:**
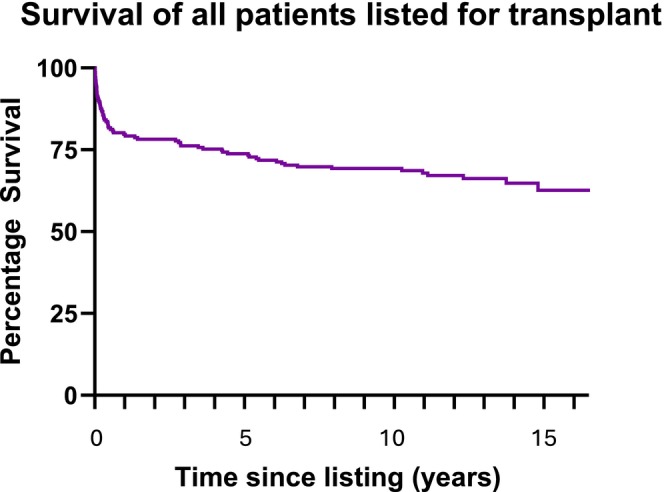
Kaplan–Meier—Survival of all patients listed for transplant. Figure showing survival of all patients after listing until end of outcome follow‐up (1st November 2024). Survival declines with time with particular decrease within the first‐year post‐transplant.

**FIGURE 4:**
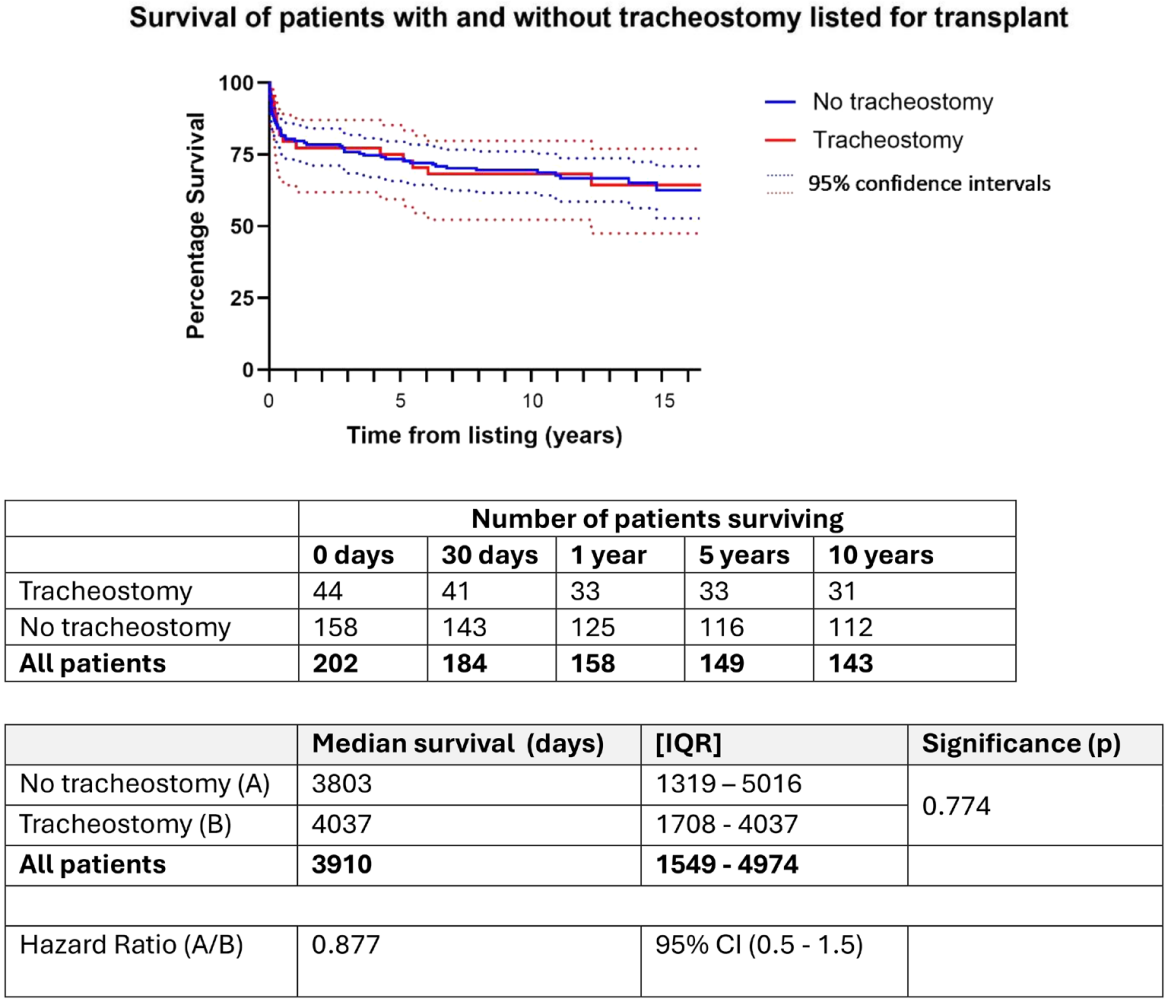
Kaplan Meier—Survival with and without tracheostomy. Figure showing survival comparison of patients with and without tracheostomy following listing for transplantation with red indicating those with tracheostomy and blue indicating those without tracheostomy. Dotted lines show 95% confidence intervals for each group. There is no statistical difference in survival between the two groups although 95% confidence intervals are broader for the tracheostomy group.

As this is the first UK study to analyze survival after pediatric cardiac transplantation with tracheostomy, it is highly significant that our study shows no difference in survival for patients who underwent tracheostomy during the transplant journey (see Kaplan–Meier survival plots, Figures 3 and 4). This was true even when following up patients at 1‐year post‐transplant, with 97% survival in non‐tracheostomy versus 94% in tracheostomy patients. This suggests that tracheostomy provides a safe means to progress and support some of the most complex patients who are listed for cardiac transplantation. It should be noted however that our sample size is small, making data more challenging to interpret.

Previous studies into tracheostomy use have suggested worse outcomes for patients following tracheostomy after cardiac surgery in comparison to other patient groups [9, 12]. Two previous UK studies have included analysis of cardiac transplant patients undergoing tracheostomy as part of a larger cohort [2, 9]. Hoskote et al. [2] studied 37 children who underwent tracheostomy following cardiothoracic surgery over a 3‐year period from 1998 to 2001. Six of these children had a cardiac transplantation with survival of 67% (4/6 patients), lower than our survival (though very small number). Roberts et al. [9] recently published a cohort of children who underwent tracheostomy in our institution over a similar time period to our study (2010–2018). They analyzed the cardiac subgroup of patients and included transplantation within this analysis. Thirty eight of 172 tracheostomies (22%) were performed on cardiac patients and mortality was higher than population with tracheostomy for other indications at 31% (9/29; follow‐up information only available for 29 patients) as compared with 22.8% (29/127). It is interesting to note the higher mortality in cardiac patients compared to other indications. Our study suggests that this difference is likely due to the highly complex and unstable nature of cardiac patients who require tracheostomy. Tracheostomy should be seen as a positive step towards progress and even recovery in cardiac transplant patients rather than a poor prognostic marker.

Spinner et al. [4] recently detailed tracheostomy usage in 74 patients (from a total of 2603 cardiac transplants) looking at patients < 21 years of age undergoing cardiac transplantation over an 11‐year period. They found an increased mortality in patients who underwent tracheostomy versus those who did not (20% vs. 5%) with a suggestion of better survival in those where tracheostomy was inserted on an earlier hospital admission, with mortality comparable to non‐tracheostomy transplant patients. It is interesting that their data suggest similar survival for those where tracheostomy was performed on a previous hospital admission. It highlights the need for international collaboration and research to better understand comparative survival in these patients.

### 4.3 Characteristics and Prevalence

Our study showed no significant differences in the demographics of the two patient groups but a trend towards lower weight and younger age. There also appeared to be an increased likelihood of tracheostomy in patients with single ventricle circulation and a decreased likelihood in those with cardiomyopathy/myocarditis. This would seem understandable given single ventricle circulation patients are more complex (with more comorbidities and genetic abnormalities) and will have undergone surgery (potentially multiple times) prior to listing, giving significantly greater scope for post‐operative complications such as vocal nerve palsy, diaphragmatic palsy as well as airway anomalies following multiple intubations. They will also be a generally younger/lower weight cohort of patients.

Prevalence of tracheostomy was high in our transplant cohort with 22% (44/202) of patients undergoing tracheostomy after listing. UK data shows overall tracheostomy rates nationally of 0.97% during PIC admission (for any indication), but significant disparity between centres with highest rate of 4% [10]. This difference across centres is likely due to the specialist services offered at some hospitals. Spinner et al. (2020) [4] found a prevalence of 2.8% requiring tracheostomy, which is comparable with another multi‐centre American study looking at tracheostomy prevalence following pediatric cardiac surgery [15] with rates across the hospitals of 0.3%–2.5%. The patients in our study were much younger at time of listing for transplantation, with Spinner et al. giving a median age of 5.1 years (IQR 0.76–13.2) whilst our median age was 2.9 (IQR 0.7–8.8). A younger cohort provides different challenges and potentially more airway issues necessitating tracheostomy. Younger patients may require more sedation with resultant increased risk of critical illness myopathy. The impact of an enlarged heart (either because of cardiomyopathy or size‐mismatch transplanted organ) has a bigger comparative effect on smaller patients due to resultant airway compression. It should be noted again that there are significant differences in the healthcare system between the UK and US and this may affect timing of listing for transplantation, patient's clinical status at listing and other variables. In the UK, if a small baby requires some form of long‐term support, tracheostomy and long‐term ventilation would frequently be considered first‐line, prior to VAD. This is because, if they achieve stability with tracheostomy/ventilation, there is the potential for de‐escalation from PIC and high dependency settings and even the possibility of time at home. This is not possible currently in the UK with a VAD in situ. The only UK study detailing tracheostomy in pediatric cardiac patients is a single centre study at our centre [9]. Unfortunately, Roberts et al. had to exclude some of these patients from analysis due to return to local hospitals from our tertiary centre [8]. In total, 38 tracheostomies were performed in pediatric cardiac patients in 8 years, mean 4.75 per year however no prevalence is given. We have included all patients listed for transplantation, many of whom did not ultimately undergo cardiac transplant. This may also account for some variation as patients listed for cardiac transplantation are, by definition, a group of patients who are not expected to recover without transplantation unlike those having routine cardiac surgery who are likely to make a full recovery after corrective surgery. We note the significant difference in waiting times for pediatric cardiac transplant in the UK versus US with UK median waiting time 193 days versus 71 days in the US [16, 17].

### 4.4 Complications

Early complications after insertion of tracheostomy were minor and infrequent with 27 (61%) patients having no reported issues. This is comparable with other literature [8, 14]. All children with tracheostomy underwent ENT follow‐up. However, we did not specifically analyze ENT outcomes within our cohort. There are additional, longer‐term complications associated with a tracheostomy, such as tracheocutaneous fistula and suprastoma collapse, which it is important to be aware of when considering tracheostomy and counseling parents. This was not assessed in our cohort, but rates are well documented in the literature [9, 11, 18].

### 4.5 Mechanical Support

It is interesting to note the slight increased prevalence of mechanical cardiac support in tracheostomy patients versus non‐tracheostomy (72% vs. 63%). This likely reflects the generally poor clinical condition of this group of patients. However, given overall mortality is not different, it suggests that tracheostomy provides favorable additional support to this challenging population. This highlights the importance of tracheostomy as it can be instigated in patients who are awaiting transplants before, after or instead of VAD support, depending on specific patient factors, to provide optimum mechanical support in severe cardiac failure.

### 4.6 Indication for Tracheostomy

There was often no specific indication for tracheostomy insertion given within the clinical notes except “failure to extubate/wean from ventilation.” Some of these patients had airway anomalies and/or phrenic nerve palsy. It is possible that these complications were a factor in the indication for tracheostomy, but this was not stated by the clinical team. We know that children with heart failure have multiple factors influencing their inability to wean from ventilation including poor cardiac function, fluid overload, muscle weakness, airway anomalies and phrenic nerve palsy. The lack of one clear indication for tracheostomy in many cases highlights the complexity of these patients and often multi‐factorial nature of their failure to wean from ventilation.

### 4.7 Airway Anomalies

It is expected that patients undergoing tracheostomy will more commonly have airway anomalies. Despite this often not being listed in the indication for tracheostomy, it seems likely that this was a factor in the need for tracheostomy in these patients given the large proportion of patients with anomalies identified at bronchoscopy (64%). Previous studies of tracheostomy use post‐cardiac surgery have similar findings.

### 4.8 Pre versus Post‐Transplant Tracheostomy

Spinner et al. [4] suggested that patients who underwent tracheostomy pre‐transplantation had better outcome than those with tracheostomy insertion post‐transplant, particularly when tracheostomy was inserted on a prior hospital visit. There is also evidence in the literature that earlier tracheostomy insertion leads to more favorable outcomes following cardiac surgery [13, 15, 19] and in critical illness [20]. From our cohort, 33 tracheostomised patients ultimately underwent cardiac transplantation. Of those, 16 patients (48%) underwent tracheostomy pre‐transplantation (note 3 prior to listing) with mortality of 31% (5/16) whilst 17 (52%) had tracheostomy inserted post‐transplantation with mortality of 24% (4/17). This suggests a slightly worse outcome for those undergoing tracheostomy pre‐transplantation in our cohort. However, given the small numbers involved, this is not a statistically significant difference, and we would suggest timing of tracheostomy around transplantation should remain a decision individualized to the patient.

### 4.9 Limitations

Our study is observational and retrospective. Complete demographic data were not found for nine patients. Some data were difficult to obtain and required analysis of individual patient notes to clarify. Despite this, it was still often not possible to obtain detail on indications for tracheostomy beyond “failure to wean from ventilation.” The authors suggest this highlights the complex and multi‐factorial nature of these cases. An assumption was made that if no complications were listed then there were none however this may not have been correctly entered within the patient record. It should also be noted that we did not specifically follow‐up patients from an ENT perspective and therefore do not have information regarding longer term complications following tracheostomy insertion.

It is important to emphasize that this study lacks power due to the small number of patients within our cohort, despite analyzing 11 years of data. This reflects the relatively small number of pediatric patients listed for transplant in a single centre. Whilst there appears to be no outcome difference between the non‐tracheostomy and tracheostomy groups, it is not possible to make definitive conclusions given the small size of the subgroup of patients requiring tracheostomy.

## 5 Conclusion

Children with end‐stage heart failure often require additional circulatory and/or respiratory support. There is no evidence from our data that the subgroup requiring tracheostomy have a reduced survival compared to children who do not. Tracheostomy is usually a short‐term measure, and most patients can be decannulated within 3 months of transplant. Further international collaborative research is needed to analyze this group more comprehensively and determine if there is indeed no difference in outcome as well as any characteristics at listing that predispose towards requirement for tracheostomy.

## Conflicts of Interest

The authors declare no conflicts of interest.

## Supporting information


**Data S1.** Supporting Information.

**Table S1.** Survival after listing, including subgroups.

**Table S2.** Statistical comparison for subgroup survival. Comparison of survival rate according to intervention using Fisher’s Exact Test.

## Data Availability

The data that support the findings of this study are available from the corresponding author upon reasonable request.
