# Supplementary material for: Tracheostomy as a Management Option After Listing for Pediatric Cardiac Transplantation
Source: Pediatr Transplant. 2025 Jan 21;29(1):e70029. doi: 10.1111/petr.70029 (PMC11750632; doi:10.1111/petr.70029)
Supplement: Supplementary file 1 — Data S1. Supporting Information. [file PETR-29-e70029-s003.docx]

**Appendices**

**Supplementary tables**

Supplementary table 1: Survival after listing, including subgroups

|  | **Transplant with tracheostomy**  (n = 33) | | **Transplant with no tracheostomy**  (n = 126) | | **No transplant with tracheostomy**  (n = 11) | | **No transplant, no tracheostomy**  (n = 32) | | **Significance** | **All patients**  (n = 202) | |
| --- | --- | --- | --- | --- | --- | --- | --- | --- | --- | --- | --- |
|  | n | (%) | n | (%) | n | (%) | n | (%) | p | n | (%) |
| **Survival Status** | | | | | | | | | | | |
| **Survived to 30 days post listing** | 31 | (94) | 121 | (96) | 10 | (91) | 22 | (69) | 0.000028 | 184 | (91) |
| **Survived to 1 year post listing** | 28 | (85) | 114 | (90) | 5 | (45) | 11 | (34) | <0.00001 | 158 | (78) |
| **Surviving at follow-up** | 24 | (73) | 96 | (76) | 2 | (18) | 9 | (28) | <0.00001 | 131 | (65) |

n = number of patients

This table details survival at 30 days post-listing, 1-year post-listing and at follow-up (on 1^st^ November 2024) for four subgroups: transplant with tracheostomy, transplant without tracheostomy, no transplant with tracheostomy and no transplant and no tracheostomy. P values are given to compare the four subgroups showing a significant difference in outcomes across the four. Supplementary table 2 provides a more detailed statistical comparison.

Supplementary table 2: Statistical comparison for subgroup survival. Comparison of survival rate according to intervention using Fisher’s Exact Test

Groups are labelled for ease of viewing:

A: Transplant with tracheostomy B: Transplant no tracheostomy

C: No transplant with tracheostomy D: No transplant, no tracheostomy

Survival of patients to 30 days post listing according to intervention group

|  | | B | | C | | D | |
| --- | --- | --- | --- | --- | --- | --- | --- |
|  |  | Transplant no tracheostomy | | No transplant with tracheostomy | | No transplant, no tracheostomy | |
|  |  | Significance (p) | Relative Risk  (95% confidence interval) | Significance (p) | Relative Risk (95% confidence interval) | Significance (p) | Relative Risk  (95% confidence interval) |
| A | Transplant with tracheostomy | 0.6355 | 1.527  (0.3 - 7.5) | 1 | 0.7  (0.07 - 6.7) | **0.0112** | 0.2  (0.1 - 0.8) |
| B | Transplant no tracheostomy |  | | **0** | 0.4  (0.1 - 3.4) | **0.0001** | 0.1  (0.1 - 0.3) |
| C | No transplant with tracheostomy |  | | | | 0.237 | 0.3  (0.1 - 2.0) |

This table details and compares the subgroups in more detail than supplementary table 1 to compare each individual subgroup with each other at 30 days post-listing. P values show statistical significance in outcome for both transplant with and without tracheostomy in comparison to those who do not have transplant or tracheostomy.

Survival of patients to 1 year post listing according to intervention group

|  | | B | | C | | D | |
| --- | --- | --- | --- | --- | --- | --- | --- |
|  |  | Transplant no tracheostomy (B) | | No transplant with tracheostomy (C) | | No transplant, no tracheostomy (D) | |
|  |  | Significance (p) | Relative Risk  (95% confidence interval) | Significance (p) | Relative Risk  (95% confidence interval) | Significance (p) | Relative Risk  (95% confidence interval) |
| A | Transplant with tracheostomy | 0.3513 | 1.6  (0.6 -4.2) | **0.0162** | 0.3  (0.1-0.7) | 0.1497 | 0.5  (0.2-1.3) |
| B | Transplant no tracheostomy |  | | **0.0007** | 0.2  (0.1 - 0.4) | **<0.00001** | 0.1  (0.1-0.3) |
| C | No transplant with tracheostomy |  | | | | 0.719 | 0.8  (0.5 -1.5) |

This table details and compares the subgroups in more detail than supplementary table 1 to compare each individual subgroup with each other at 1-year post-listing. P values show statistical significance in outcome for transplant without tracheostomy in comparison to no transplant with or without tracheostomy. There is also statistical significance seen for those with transplant and tracheostomy in comparison to no transplant and tracheostomy although no significant different to those who do not have transplant or tracheostomy.

Survival of patients to follow-up according to intervention group

|  | | B | | C | | D | |
| --- | --- | --- | --- | --- | --- | --- | --- |
|  |  | Transplant no tracheostomy (B) | | No transplant with tracheostomy (C) | | No transplant, no tracheostomy (D) | |
|  |  | Significance (p) | Relative Risk  (95% confidence interval) | Significance (p) | Relative Risk  (95% confidence interval) | Significance (p) | Relative Risk  (95% confidence interval) |
| A | Transplant with tracheostomy | 0.6564 | 1.5  (0.6 -2.2) | **0.0032** | 0.3  (0.2-0.6) | **0.0005** | 0.4  (0.2-0.7) |
| B | Transplant no tracheostomy |  | | **0.0002** | 0.3  (0.2 - 0.4) | **<0.00001** | 0.3  (0.2-0.5) |
| C | No transplant with tracheostomy |  | | | | 0.6983 | 1.1  (0.8 -1.6) |

This table details and compares the subgroups in more detail than supplementary table 1 to compare each individual subgroup with each other at follow-up (1^st^ November 2024). P values show statistical significance in outcome for both transplant with and without tracheostomy in comparison to no transplant with or without tracheostomy.
