# Supplementary material for: Tracheostomy as a Management Option After Listing for Pediatric Cardiac Transplantation
Source: Pediatr Transplant. 2025 Jan 21;29(1):e70029. doi: 10.1111/petr.70029 (PMC11750632; doi:10.1111/petr.70029)
Supplement: Supplementary file 2 — Table S1. Survival after listing, including subgroups. [file PETR-29-e70029-s001.pdf]

Supplementary table 1: Survival after listing, including subgroups

	**Transplant with tracheostomy** (n = 33)		**Transplant with no tracheostomy** (n = 126)		**No transplant with tracheostomy** (n = 11)		**No transplant, no tracheostomy** (n = 32)		**Significance**	**All patients** (n = 202)	
	n	(%)	n	(%)	n	(%)	n	(%)	p	n	(%)
**Survival Status**											
**Survived to 30 days post listing**	31	(94)	121	(96)	10	(91)	22	(69)	0.000028	184	(91)
**Survived to 1 year post listing**	28	(85)	114	(90)	5	(45)	11	(34)	<0.00001	158	(78)
**Surviving at follow-up**	24	(73)	96	(76)	2	(18)	9	(28)	<0.00001	131	(65)

n = number of patients

This table details survival at 30 days post-listing, 1-year post-listing and at follow-up (on 1st November 2024) for four subgroups: transplant with tracheostomy, transplant without tracheostomy, no transplant with tracheostomy and no transplant and no tracheostomy. P values are given to compare the four subgroups showing a significant difference in outcomes across the four. Supplementary table 2 provides a more detailed statistical comparison.
