# Supplementary material for: Tracheostomy as a Management Option After Listing for Pediatric Cardiac Transplantation
Source: Pediatr Transplant. 2025 Jan 21;29(1):e70029. doi: 10.1111/petr.70029 (PMC11750632; doi:10.1111/petr.70029)
Supplement: Supplementary file 3 — Table S2. Statistical comparison for subgroup survival. Comparison of survival rate according to intervention using Fisher’s Exact Test. [file PETR-29-e70029-s002.pdf]

Supplementary table 2: Statistical comparison for subgroup survival. Comparison of survival rate according to intervention using Fisher's Exact Test

Groups are labelled for ease of viewing:

A: Transplant with tracheostomy

B: Transplant no tracheostomy

C: No transplant with tracheostomy

D: No transplant, no tracheostomy

Tables show statistical analysis of survival data shown in supplementary table 1. Each group is compared with another for analysis.

Survival of patients to 30 days post listing according to intervention group

		B		C		D	
		Transplant no tracheostomy		No transplant with tracheostomy		No transplant, no tracheostomy	
		Significance (p)	Relative Risk (95% confidence interval)	Significance (p)	Relative Risk (95% confidence interval)	Significance (p)	Relative Risk (95% confidence interval)
A	Transplant with tracheostomy	0.6355	1.527 (0.3 - 7.5)	0.7299	0.7 (0.07 - 6.7)	**0.0112**	0.2 (0.1 - 0.8)
B	Transplant no tracheostomy			0.4296	0.4 (0.1 - 3.4)	**0.0001**	0.1 (0.1 - 0.3)
C	No transplant with tracheostomy					0.237	0.3 (0.1 - 2.0)

This table details and compares the subgroups in more detail than supplementary table 1 to compare each individual subgroup with each other at 30 days post-listing. P values show statistical significance in outcome for both transplant with and without tracheostomy in comparison to those who do not have transplant or tracheostomy.

Survival of patients to 1 year post listing according to intervention group

		B		C		D	
		Transplant no tracheostomy (B)		No transplant with tracheostomy (C)		No transplant, no tracheostomy (D)	
		Significance (p)	Relative Risk (95% confidence interval)	Significance (p)	Relative Risk (95% confidence interval)	Significance (p)	Relative Risk (95% confidence interval)
A	Transplant with tracheostomy	0.3513	1.6 (0.6 -4.2)	**0.0162**	0.3 (0.1-0.7)	0.1497	0.5 (0.2-1.3)
B	Transplant no tracheostomy			**0.0007**	0.2 (0.1 - 0.4)	**<0.00001**	0.1 (0.1-0.3)
C	No transplant with tracheostomy					0.719	0.8 (0.5 -1.5)

This table details and compares the subgroups in more detail than supplementary table 1 to compare each individual subgroup with each other at 1-year post-listing. P values show statistical significance in outcome for transplant without tracheostomy in comparison to no transplant with or without tracheostomy. There is also statistical significance seen for those with transplant and tracheostomy in comparison to no transplant and tracheostomy although no significant different to those who do not have transplant or tracheostomy.

Survival of patients to follow-up according to intervention group

		B		C		D	
		Transplant no tracheostomy (B)		No transplant with tracheostomy (C)		No transplant, no tracheostomy (D)	
		Significance (p)	Relative Risk (95% confidence interval)	Significance (p)	Relative Risk (95% confidence interval)	Significance (p)	Relative Risk (95% confidence interval)
A	Transplant with tracheostomy	0.6564	1.5 (0.6 -2.2)	**0.0032**	0.3 (0.2-0.6)	**0.0005**	0.4 (0.2-0.7)
B	Transplant no tracheostomy			**0.0002**	0.3 (0.2 - 0.4)	**<0.00001**	0.3 (0.2-0.5)
C	No transplant with tracheostomy					0.6983	1.1 (0.8 -1.6)

This table details and compares the subgroups in more detail than supplementary table 1 to compare each individual subgroup with each other at follow-up (1st November 2024). P values show statistical significance in outcome for both transplant with and without tracheostomy in comparison to no transplant with or without tracheostomy.
